# Tetra­aqua­bis­[2-(4-pyridyl­sulfan­yl)acetato-κ*N*]nickel(II)

**DOI:** 10.1107/S1600536811019131

**Published:** 2011-05-25

**Authors:** Jing-Yi Wang, Xiu-Guang Wang, Xiao-Jun Zhao

**Affiliations:** a College of Chemistry, Tianjin Key Laboratory of Structure and Performance for Functional Molecules, Tianjin Normal University, Tianjin 300387, People’s Republic of China

## Abstract

In the centrosymmetric title complex, [Ni(C_7_H_6_NO_2_S)_2_(H_2_O)_4_], the Ni^II^ atom, located on a centre of inversion, is coordinated by two N atoms from two 2-(4-pyridyl­sulfan­yl)acetate ligands and four water O atoms in an octa­hedral geometry. In the crystal, inter­molecular O—H⋯O hydrogen bonds between the coordinated water mol­ecules and the carboxyl­ate group of the anionic 2-(4-pyridyl­sulfan­yl)acetate ligands link these discrete mononuclear units into a three-dimensional network.

## Related literature

For structures and applications of metal complexes with polycarboxyl­ate-based pyridine ligands, see: Zhao *et al.* (2010 ▶); Wang *et al.* (2007 ▶). For metal complexes with 2-(4-pyridyl­sulfan­yl)acetate ligands, see: Kondo *et al.* (2002 ▶); Zhang *et al.* (2004 ▶); Qin *et al.* (2004 ▶).
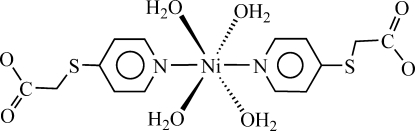


## Experimental

### 

#### Crystal data

[Ni(C_7_H_6_NO_2_S)_2_(H_2_O)_4_]
*M* *_r_* = 467.15
Triclinic, 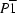

*a* = 6.3577 (4) Å
*b* = 7.0330 (5) Å
*c* = 11.7624 (8) Å
α = 92.713 (1)°
β = 103.440 (1)°
γ = 115.120 (1)°
*V* = 456.75 (5) Å^3^
*Z* = 1
Mo *K*α radiation
μ = 1.34 mm^−1^
*T* = 296 K
0.22 × 0.20 × 0.14 mm

#### Data collection

Bruker APEXII CCD area-detector diffractometer
Absorption correction: multi-scan (*SADABS*; Bruker, 2001 ▶) *T*_min_ = 0.758, *T*_max_ = 0.835
2358 measured reflections
1610 independent reflections
1525 reflections with *I* > 2σ(*I*)
*R*_int_ = 0.008

#### Refinement

*R*[*F* ^2^ > 2σ(*F* ^2^)] = 0.022
*wR*(*F* ^2^) = 0.061
*S* = 1.05
1610 reflections
124 parameters
H-atom parameters constrained
Δρ_max_ = 0.22 e Å^−3^
Δρ_min_ = −0.28 e Å^−3^

### 

Data collection: *APEX2* (Bruker, 2003 ▶); cell refinement: *SAINT* (Bruker, 2001 ▶); data reduction: *SAINT*; program(s) used to solve structure: *SHELXS97* (Sheldrick, 2008 ▶); program(s) used to refine structure: *SHELXL97* (Sheldrick, 2008 ▶); molecular graphics: *SHELXTL* (Sheldrick, 2008 ▶) and *DIAMOND* (Brandenburg & Berndt, 1999 ▶); software used to prepare material for publication: *SHELXL97*.

## Supplementary Material

Crystal structure: contains datablocks I, global. DOI: 10.1107/S1600536811019131/bt5549sup1.cif

Structure factors: contains datablocks I. DOI: 10.1107/S1600536811019131/bt5549Isup2.hkl

Additional supplementary materials:  crystallographic information; 3D view; checkCIF report

## Figures and Tables

**Table 1:** Hydrogen-bond geometry (Å, °)

*D*—H⋯*A*	*D*—H	H⋯*A*	*D*⋯*A*	*D*—H⋯*A*
O3—H3*A*⋯O2^i^	0.85	2.13	2.951 (2)	161
O3—H3*B*⋯O1^ii^	0.85	1.92	2.7265 (18)	158
O4—H4*A*⋯O1^iii^	0.85	1.83	2.6697 (18)	168
O4—H4*B*⋯O2^iv^	0.85	2.01	2.855 (2)	172

Symmetry codes: (i) 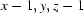; (ii) 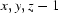; (iii) 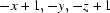; (iv) 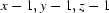.

**Fig. 1.**
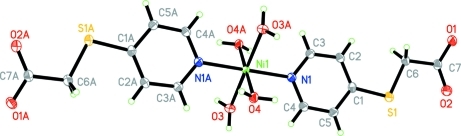
The molecular structure of the title compound. Displacement ellipsoids are drawn at the 30% probablity level. [Symmetry code: (A) –x, –y, –z.]

**Fig. 2.**
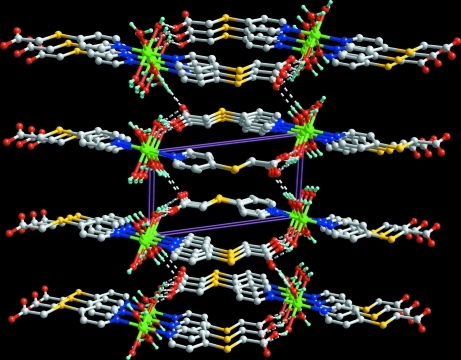
Crystal packing of the title compound, showing O—H···O hydrogen bonds as dashed lines. Only H atoms involved in hydrogen bonds have been included.

## supplementary crystallographic information

### Comment

Recently, functional metal complexes with polycarboxylate-based pyridine ligands have gained more and more interest due to their intriguing structures and potential applications in magnetism (Zhao *et al.* 2010), and luminescence (Wang *et al.* 2007). Acting as one of flexible multifunctional building blocks, 2-(4-pyridylsulfanyl)acetic acid with three potential metal binding sites and various flexible connection modes (Kondo *et al.*, 2002; Zhang *et al.*, 2004; Qin *et al.*, 2004) has been extensively used to construct novel metal complexes with discrete mononuclear structure or polymeric coordination framework with variable dimensionality. Herein, as the continuing investigations on the coordination chemistry of the ligand, we report the crystal structure of a tetraaquonickel(II) complex with deprotonated 2-(4-pyridylsulfanyl)acetate ligand, (I).

The molecular structure of the title mononuclear complex is show Fig.1 and selected bond lengths and angles are listed in Table 1. The Ni^II^ atom in the mononuclear structure of I lies on an inversion centre and is in a octahedral coordination environment involving two pyridyl N atoms from two different 2-(4-pyridylsulfanyl)acetate ligand and four O donors from four water molecules. In the crystal structure, four intermolecular O—H···O hydrogen bonds between the coordinated water molecules and the carboxylate group of 2-(4-pyridylsulfanyl)acetate ligand link adjacent mononuclear structures into a three-dimensional supramolecular network (Fig.2 and Table 2).

### Experimental

A methanol solution of 2-(4-pyridylsulfanyl)acetic acid (25.3 mg, 0.1 mmol) was carefully layered onto a buffer layer of ethyl acetate (2.0 ml) in a straight glass tube, meanwhile the pH value of the top layer was carefully adjusted to 7.0 by slow addition of triethylamine. Below which an aqueous solution containing NiCl_2_.6H_2_O (23.7 mg, 0.1 mmol) was placed. The test tube was left in air under room temperature. Blue block-shaped crystals were harvested within three weeks. Yield: 50% based on 2-(4-pyridylsulfanyl)acetic acid. Anal. Calcd. for C_14_H_20_N_2_NiO_8_S_2_: C, 36.00; H, 4.32; N, 6.00%. Found: C, 35.98; H, 4.34; N, 6.03%.

### Refinement

H atoms were located in a difference map, but refined using a riding model with C_aromatic_-H = 0.93Å, C_methylene_-H = 0.97Å or O-H = 0.85Å. U(H) was set to 1.2 U_eq_(C) or 1.5 U_eq_(O).

### Crystal data

**Table:**

[Ni(C_7_H_6_NO_2_S)_2_(H_2_O)_4_]	*Z* = 1
*M**_r_* = 467.15	*F*(000) = 242
Triclinic, *P*1	*D*_x_ = 1.698 Mg m^−^^3^
Hall symbol: -P 1	Mo *K*α radiation, λ = 0.71073 Å
*a* = 6.3577 (4) Å	Cell parameters from 2208 reflections
*b* = 7.0330 (5) Å	θ = 3.2–27.8°
*c* = 11.7624 (8) Å	µ = 1.34 mm^−^^1^
α = 92.713 (1)°	*T* = 296 K
β = 103.440 (1)°	Block, blue
γ = 115.120 (1)°	0.22 × 0.20 × 0.14 mm
*V* = 456.75 (5) Å^3^	

### Data collection

**Table:**

Bruker APEXII CCD area-detector diffractometer	1610 independent reflections
Radiation source: fine-focus sealed tube	1525 reflections with *I* > 2σ(*I*)
graphite	*R*_int_ = 0.008
φ and ω scans	θ_max_ = 25.0°, θ_min_ = 1.8°
Absorption correction: multi-scan (*SADABS*; Bruker, 2001)	*h* = −7→7
*T*_min_ = 0.758, *T*_max_ = 0.835	*k* = −5→8
2358 measured reflections	*l* = −13→13

### Refinement

**Table:**

Refinement on *F*^2^	Primary atom site location: structure-invariant direct methods
Least-squares matrix: full	Secondary atom site location: difference Fourier map
*R*[*F*^2^ > 2σ(*F*^2^)] = 0.022	Hydrogen site location: inferred from neighbouring sites
*wR*(*F*^2^) = 0.061	H-atom parameters constrained
*S* = 1.05	*w* = 1/[σ^2^(*F*_o_^2^) + (0.0331*P*)^2^ + 0.2312*P*] where *P* = (*F*_o_^2^ + 2*F*_c_^2^)/3
1610 reflections	(Δ/σ)_max_ = 0.001
124 parameters	Δρ_max_ = 0.22 e Å^−^^3^
0 restraints	Δρ_min_ = −0.28 e Å^−^^3^

### Special details

**Table:**

Geometry. All e.s.d.'s (except the e.s.d. in the dihedral angle between two l.s. planes) are estimated using the full covariance matrix. The cell e.s.d.'s are taken into account individually in the estimation of e.s.d.'s in distances, angles and torsion angles; correlations between e.s.d.'s in cell parameters are only used when they are defined by crystal symmetry. An approximate (isotropic) treatment of cell e.s.d.'s is used for estimating e.s.d.'s involving l.s. planes.
Refinement. Refinement of *F*^2^ against ALL reflections. The weighted *R*-factor *wR* and goodness of fit *S* are based on *F*^2^, conventional *R*-factors *R* are based on *F*, with *F* set to zero for negative *F*^2^. The threshold expression of *F*^2^ > σ(*F*^2^) is used only for calculating *R*-factors(gt) *etc*. and is not relevant to the choice of reflections for refinement. *R*-factors based on *F*^2^ are statistically about twice as large as those based on *F*, and *R*- factors based on ALL data will be even larger.

### Fractional atomic coordinates and isotropic or equivalent isotropic displacement parameters (Å^2^)

**Table:**

	*x*	*y*	*z*	*U*_iso_*/*U*_eq_	
Ni1	0.0000	0.0000	0.0000	0.02365 (11)	
S1	0.72786 (9)	0.33945 (8)	0.54727 (4)	0.03489 (14)	
O1	0.5787 (3)	0.2690 (3)	0.84929 (12)	0.0430 (4)	
O2	0.9194 (3)	0.4387 (2)	0.79704 (12)	0.0423 (4)	
O3	0.2259 (2)	0.2716 (2)	−0.05684 (11)	0.0347 (3)	
H3A	0.1377	0.3317	−0.0833	0.052*	
H3B	0.3544	0.3065	−0.0783	0.052*	
O4	0.1770 (2)	−0.1742 (2)	−0.03636 (11)	0.0302 (3)	
H4A	0.2585	−0.1876	0.0284	0.045*	
H4B	0.0891	−0.2861	−0.0878	0.045*	
N1	0.2203 (3)	0.1150 (2)	0.17292 (13)	0.0275 (3)	
C1	0.5198 (3)	0.2521 (3)	0.40628 (15)	0.0268 (4)	
C2	0.2691 (3)	0.1584 (3)	0.38241 (16)	0.0307 (4)	
H2	0.1970	0.1401	0.4440	0.037*	
C3	0.1292 (3)	0.0933 (3)	0.26627 (16)	0.0315 (4)	
H3	−0.0381	0.0307	0.2517	0.038*	
C4	0.4625 (3)	0.2085 (3)	0.19676 (16)	0.0313 (4)	
H4	0.5298	0.2269	0.1334	0.038*	
C5	0.6160 (3)	0.2784 (3)	0.30962 (16)	0.0312 (4)	
H5	0.7827	0.3427	0.3216	0.037*	
C6	0.5391 (3)	0.2753 (3)	0.64667 (15)	0.0301 (4)	
H6A	0.4421	0.3520	0.6348	0.036*	
H6B	0.4304	0.1240	0.6296	0.036*	
C7	0.6951 (3)	0.3354 (3)	0.77521 (16)	0.0306 (4)	

### Atomic displacement parameters (Å^2^)

**Table:**

	*U*^11^	*U*^22^	*U*^33^	*U*^12^	*U*^13^	*U*^23^
Ni1	0.02087 (17)	0.03278 (19)	0.01705 (17)	0.01169 (14)	0.00582 (12)	0.00240 (12)
S1	0.0305 (3)	0.0442 (3)	0.0201 (2)	0.0099 (2)	0.00367 (18)	0.0011 (2)
O1	0.0413 (8)	0.0722 (10)	0.0279 (7)	0.0330 (8)	0.0153 (6)	0.0169 (7)
O2	0.0346 (8)	0.0547 (9)	0.0266 (7)	0.0118 (7)	0.0058 (6)	0.0018 (6)
O3	0.0292 (7)	0.0405 (7)	0.0353 (7)	0.0138 (6)	0.0139 (6)	0.0101 (6)
O4	0.0297 (6)	0.0415 (7)	0.0210 (6)	0.0189 (6)	0.0049 (5)	0.0013 (5)
N1	0.0264 (8)	0.0353 (8)	0.0218 (7)	0.0146 (7)	0.0075 (6)	0.0035 (6)
C1	0.0321 (9)	0.0247 (8)	0.0219 (9)	0.0131 (7)	0.0044 (7)	0.0015 (7)
C2	0.0327 (10)	0.0376 (10)	0.0221 (9)	0.0146 (8)	0.0104 (7)	0.0042 (8)
C3	0.0272 (9)	0.0424 (11)	0.0244 (9)	0.0147 (8)	0.0081 (8)	0.0039 (8)
C4	0.0304 (10)	0.0406 (10)	0.0228 (9)	0.0146 (8)	0.0103 (7)	0.0028 (8)
C5	0.0254 (9)	0.0374 (10)	0.0270 (9)	0.0115 (8)	0.0066 (7)	0.0011 (8)
C6	0.0337 (10)	0.0320 (9)	0.0237 (9)	0.0143 (8)	0.0070 (8)	0.0055 (7)
C7	0.0361 (10)	0.0358 (10)	0.0233 (9)	0.0198 (9)	0.0073 (8)	0.0041 (7)

### Geometric parameters (Å, °)

**Table:**

Ni1—O4	2.0727 (12)	N1—C3	1.342 (2)
Ni1—O4^i^	2.0727 (12)	N1—C4	1.344 (2)
Ni1—N1	2.0762 (15)	C1—C2	1.392 (3)
Ni1—N1^i^	2.0762 (15)	C1—C5	1.396 (3)
Ni1—O3^i^	2.0967 (13)	C2—C3	1.377 (3)
Ni1—O3	2.0967 (13)	C2—H2	0.9300
S1—C1	1.7524 (18)	C3—H3	0.9300
S1—C6	1.8035 (19)	C4—C5	1.373 (3)
O1—C7	1.253 (2)	C4—H4	0.9300
O2—C7	1.249 (2)	C5—H5	0.9300
O3—H3A	0.8501	C6—C7	1.528 (3)
O3—H3B	0.8503	C6—H6A	0.9700
O4—H4A	0.8501	C6—H6B	0.9700
O4—H4B	0.8501		
			
O4—Ni1—O4^i^	179.999 (1)	C2—C1—C5	117.45 (16)
O4—Ni1—N1	92.68 (5)	C2—C1—S1	125.97 (14)
O4^i^—Ni1—N1	87.32 (5)	C5—C1—S1	116.57 (14)
O4—Ni1—N1^i^	87.32 (5)	C3—C2—C1	119.08 (17)
O4^i^—Ni1—N1^i^	92.68 (5)	C3—C2—H2	120.5
N1—Ni1—N1^i^	180.0	C1—C2—H2	120.5
O4—Ni1—O3^i^	86.23 (5)	N1—C3—C2	123.81 (17)
O4^i^—Ni1—O3^i^	93.77 (5)	N1—C3—H3	118.1
N1—Ni1—O3^i^	89.32 (5)	C2—C3—H3	118.1
N1^i^—Ni1—O3^i^	90.69 (5)	N1—C4—C5	123.50 (17)
O4—Ni1—O3	93.77 (5)	N1—C4—H4	118.3
O4^i^—Ni1—O3	86.23 (5)	C5—C4—H4	118.3
N1—Ni1—O3	90.69 (5)	C4—C5—C1	119.45 (17)
N1^i^—Ni1—O3	89.31 (5)	C4—C5—H5	120.3
O3^i^—Ni1—O3	180.0	C1—C5—H5	120.3
C1—S1—C6	103.65 (9)	C7—C6—S1	110.25 (13)
Ni1—O3—H3A	105.2	C7—C6—H6A	109.6
Ni1—O3—H3B	134.1	S1—C6—H6A	109.6
H3A—O3—H3B	117.1	C7—C6—H6B	109.6
Ni1—O4—H4A	109.3	S1—C6—H6B	109.6
Ni1—O4—H4B	114.5	H6A—C6—H6B	108.1
H4A—O4—H4B	117.0	O2—C7—O1	126.41 (18)
C3—N1—C4	116.70 (16)	O2—C7—C6	119.17 (17)
C3—N1—Ni1	122.04 (12)	O1—C7—C6	114.39 (17)
C4—N1—Ni1	121.22 (12)		
			
O4—Ni1—N1—C3	−125.81 (15)	C4—N1—C3—C2	−0.8 (3)
O4^i^—Ni1—N1—C3	54.19 (15)	Ni1—N1—C3—C2	176.86 (15)
O3^i^—Ni1—N1—C3	−39.62 (15)	C1—C2—C3—N1	−0.1 (3)
O3—Ni1—N1—C3	140.38 (15)	C3—N1—C4—C5	0.8 (3)
O4—Ni1—N1—C4	51.79 (15)	Ni1—N1—C4—C5	−176.94 (14)
O4^i^—Ni1—N1—C4	−128.21 (15)	N1—C4—C5—C1	0.2 (3)
O3^i^—Ni1—N1—C4	137.98 (15)	C2—C1—C5—C4	−1.2 (3)
O3—Ni1—N1—C4	−42.02 (15)	S1—C1—C5—C4	178.42 (14)
C6—S1—C1—C2	0.69 (19)	C1—S1—C6—C7	178.07 (12)
C6—S1—C1—C5	−178.92 (14)	S1—C6—C7—O2	8.2 (2)
C5—C1—C2—C3	1.2 (3)	S1—C6—C7—O1	−170.33 (14)
S1—C1—C2—C3	−178.44 (14)		

Symmetry codes: (i) −*x*, −*y*, −*z*.

### Hydrogen-bond geometry (Å, °)

**Table:**

*D*—H···*A*	*D*—H	H···*A*	*D*···*A*	*D*—H···*A*
O3—H3A···O2^ii^	0.85	2.13	2.951 (2)	161
O3—H3B···O1^iii^	0.85	1.92	2.7265 (18)	158
O4—H4A···O1^iv^	0.85	1.83	2.6697 (18)	168
O4—H4B···O2^v^	0.85	2.01	2.855 (2)	172

Symmetry codes: (ii) *x*−1, *y*, *z*−1; (iii) *x*, *y*, *z*−1; (iv) −*x*+1, −*y*, −*z*+1; (v) *x*−1, *y*−1, *z*−1.
